# CDK4/6 inhibition with dual immunotherapy in chemorefractory SMARCA4-deficient undifferentiated tumor: a case report

**DOI:** 10.3389/fimmu.2026.1858358

**Published:** 2026-05-19

**Authors:** Ünal Metin Tokat, Şevval Nur Bilgiç, Ashkan Adibi, Esranur Aydın, Onur Tutar, Hilal Cansız, Ali Yılmaz Altay, Nalan Akgul Babacan, Eylül Özgü, Alina Melisa Manea, Mutlu Demiray

**Affiliations:** 1 Precision Oncology Center, Medicana Health Group, Istanbul, Türkiye; 2 Department of Internal Medicine, Cerrahpasa Faculty of Medicine, Istanbul University, Istanbul, Türkiye; 3 Ear Nose and Throat Clinic, Istanbul Haseki Training and Research Hospital, Sultangazi, Istanbul, Türkiye; 4 Department of Medical Pathology, Department of Surgical Medical Sciences, Istanbul Faculty of Medicine, Istanbul University, Istanbul, Türkiye

**Keywords:** Thoracic SMARCA4-deficient undifferentiated tumor (SMARCA4-UT), immunotherapy, CDK4/6 inhibitor, precision oncology, pembrolizumab, ipilimumab, palbociclib, case report

## Abstract

Thoracic SMARCA4-deficient undifferentiated tumors (SMARCA4-UT) are rare malignancies characterized by resistance to chemotherapy and poor clinical outcomes. While immunotherapy have shown promise, especially in the first-line treatment, effective therapeutic strategies for patients with PD-L1-negative tumors and complex genomic profiles remain undefined. We report a case of chemorefractory SMARCA4-UT in a patient presenting with cervical mass. CGP revealed pathogenic *SMARCA4*, *TP53*, and *CDKN2A* mutations. Despite PD-L1 negativity, the tumor exhibited TMB-H and a dominant smoking signature. Guided by precision oncology targeting both immunogenic profile and cell-cycle dysregulation, the patient was treated with dual immunotherapy (pembrolizumab plus ipilimumab) combined with the CDK4/6 inhibitor palbociclib. This novel regimen elicited a metabolic partial response within 1.5 months, which has been sustained for over 4 months. To our knowledge, this is the first report demonstrating the efficacy of dual checkpoint blockade plus CDK4/6 inhibition in SMARCA4-UT. This case highlights potential of biomarker-driven therapies to overcome resistance in rare thoracic neoplasms.

## Introduction

Thoracic SMARCA4-deficient undifferentiated tumors are ultra-rare, aggressive malignant neoplasms that predominantly affect middle-aged males (1, 2). Often diagnosed at advanced stage, they are most commonly located in the mediastinum, followed by the pleura and lung. They frequently metastasize to the adrenal glands, lymph nodes (LNs) and bone, and exhibit a strong association with heavy smoking (1). Although more than 150 cases had been reported as of December 2023 (3) and the prevalence for the older synonymous label (SMARCA4-deficient thoracic sarcoma) was listed as <1 per 1,000,000 in Orphanet, there are no well-established prevalence and incidence estimates for SMARCA4-UT as it is a newly recognized WHO entity with a potential for misdiagnosis. SMARCA4-UTs are chemorefractory with low response rates and short duration of response with the first-line chemotherapy (4).

SMARCA4 serves as a core catalytic subunit of SWI/SNF complex through its bromodomain and helicase/ATPase activity (5). These complexes are integral to transcriptional regulation, directing the specific programs that mediates differentiation and lineage commitment. SMARCA4-UT is characterized by SMARCA4 loss that is usually accompanied by SMARCA2 loss (6, 7). Genomic profiling revealed common *SMARCA4*, *TP53*, *KEAP1*, *STK11* and *KRAS* mutations alongside a high TMB and smoking signature (8).

*SMARCA4* alterations have been linked to both response and resistance to immune checkpoint inhibitors (ICIs) across diverse cancer types and contexts (9–11). There are recent reports demonstrating the efficacy of immunotherapy, usually with chemotherapy or chemoradiotherapy, in the PD-L1-positive, *CD274*-amplified and/or TMB-high SMARCA4-UTs (12–14). However, a recent study reported SMARCA4-UTs typically exhibit an immune-desert tumor microenvironment (TME), which was associated with limited clinical benefit from ICIs (15). In preclinical studies of SCCOHT (16) (plus a case report (17)) and NSCLC (18), *SMARCA4* loss or alterations were associated with sensitivity to CDK4/6 inhibitors by reducing cyclin D1. However, palbociclib or ribociclib monotherapy have deemed insufficient in advanced cancers with cyclin D-CDK4/6 pathway alterations, including complete loss of SMARCA4, in the MoST and DRUP trials (19). Furthermore, the combination of the CDK4/6 inhibitors with the ICIs has yet to be explored in the SMARCA4-UT.

To the best of our knowledge, this is the first case to report an exceptional response upon a CDK4/6 inhibitor (palbociclib) plus dual immunotherapy (pembrolizumab + ipilimumab) following precision oncology principles in a p16- and PD-L1-negative, TMB-H SMARCA4-UT.

## Case presentation and results

### Clinical history

A 37-year-old male patient with a history of hypothyroidism was referred to our clinic. He was on regular levothyroxine. He had a heavy smoking history (borderline >20 pack-years) and reported cannabis use. The patient presented with a right cervical swelling in February 2025, and was subsequently admitted to our department in March 2025. Physical examination revealed a palpable mass in the right cervical region. Panendoscopic examination of the upper aerodigestive tract revealed no pathological findings. The patient denied B symptoms. Histopathological analysis (March 2025) described an isoechoic solid nodule in the right submandibular region with a fatty appearance, loss of fatty hilum, and sparse vascularity. Microscopic examination revealed numerous single and irregular clustered atypical cells, which exhibited large hyperchromatic nuclei, scanty cytoplasm, and marked pleomorphism.

Cervical ultrasonography demonstrated multiple ovoid LNs at level II, characterized by hypoechoic and heterogeneous architecture, with both peripheral and central vascularization. Subsequent contrast-enhanced MRI revealed lesions consistent with lymphadenopathy (LAP) within the right cervical chain at level IIb, exhibiting heterogeneous signal intensity. Fine-needle aspiration (FNA) biopsy yielded a malignant cytology, with lymphoma being the primary differential diagnosis. Excisional biopsy was recommended for definitive diagnosis. Baseline (18)F-FDG PET/CT demonstrated intense FDG uptake within conglomerate LNs at the right cervical levels IB/IIB (SUVmax: 12.56). Furthermore, hypermetabolic conglomerate mediastinal LAP was identified in the right paratracheal, prevascular, and subcarinal stations (SUVmax: 15.60). Subsequently, the patient underwent a cervical excisional LN biopsy. Intraoperative exploration revealed conglomerate LN masses at the cervical levels II and III, which were adherent to surrounding tissues and caused occlusion of the internal jugular vein. These masses were surgically resected. During the postoperative follow-up, the patient developed new LAPs in the left cervical, submental, and right cervical regions.

**Figure 1:**
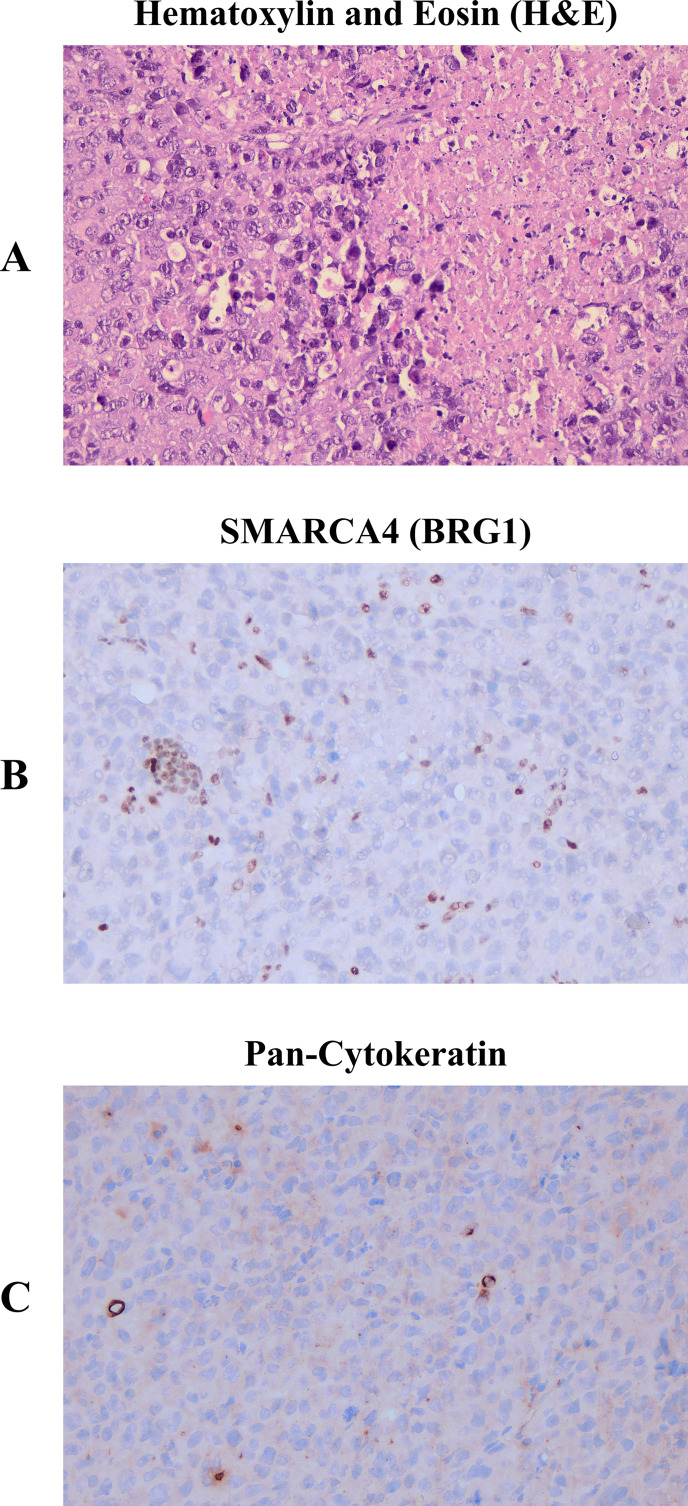
Histopathological analysis with immunohistochemistry. **(A)** High grade tumor with necrosis. Tumor cells are highly pleomorphic with visible nucleoli, H&E staining. **(B)** Tumor cells show no SMARCA4 (BRG1) expression while lymphocytes retain SMARCA4/BRG1 expression and act as internal control. **(C)** Tumor cells are negative for Pan-Cytokeratin. All images are shown at x400 magnification.

**Figure 2:**
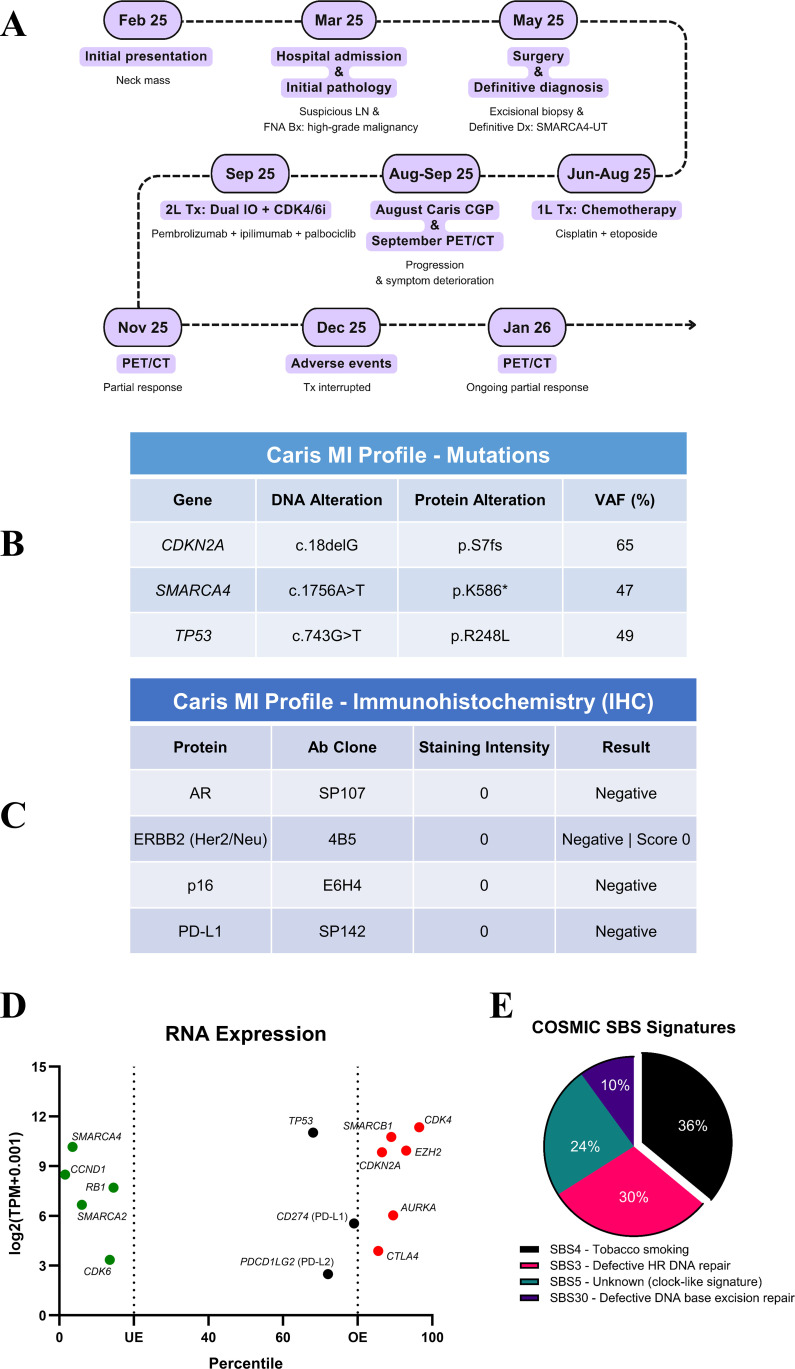
Clinical timeline of the patient and molecular characterization of the tumor. **(A)** The clinical management and treatment of the patient, starting from the initial presentation to the clinical response upon dual immunotherapy plus palbociclib. **(B-E)** Mutational profile, immunohistochemistry results, RNA expression percentiles and log_2_(TPM + 0.001) of the selected genes (UE: underexpression; OE: overexpression), and COSMIC SBS signatures are obtained through Caris MI Profile testing.

Macroscopic examination revealed a homogeneous ‘fish-flesh’ cut surface, a classic gross morphological feature highly suspicious for lymphoma. Subsequent immunohistochemistry (IHC) analyses (May 2025) reported that the tumor was diffuse positive for CD34, partially positive for EMA, and positive for LCA; whereas it was negative for SMARCA4/BRG1 (**Figure 1**), pan-Cytokeratin (**Figure 1**), CD20, CD3, MUM1, SF1, CD38, HHV8, and ALK1, with CD30 showing focal weak/negative staining. The comprehensive genomic profiling (CGP) analysis revealed a *SMARCA4* truncation mutation, explaining SMARCA4/BRG1 loss and supporting the definitive diagnosis of “thoracic SMARCA4-deficient undifferentiated tumor”. The patient was initially treated with cisplatin 75 mg/m^2^ on day 1 and etoposide 80 mg/m^2^ on days 1–3 every 21 days. However, he did not respond with a deterioration in his clinical status (**Figure 2A**).

### CGP-guided treatment

The CGP using lymph nodes of head, face and neck (Caris MI Profile, August 2025, **Figures 2B–E**, **Table 1**) revealed pathogenic *SMARCA4* K586* (Variant allele frequency, VAF: 47%), *TP53* R248L (49%) and *CDKN2A* S7fs (65%) alterations, a tumor mutational burden (TMB) of 11 muts/Mb, and stable microsatellite status. *PIK3CA* V146I (c.436G>A, 33%) VUS was considered benign through variant effect prediction analysis via GeneBe (20). Although genomic loss of heterozygosity (gLOH) was high (17%, threshold ≥16%), HRD-GSS (22) was negative when assessed by the threshold of ≥ 46. The IHC staining for PD-L1, HER2, AR and p16 were all negative. Mutational COSMIC signature analysis identified SBS4 (tobacco, 36%), SBS3 (HRD, 30%), SBS5 (24%), and SBS30 (10%) signatures.

**Table 1:** Molecular characteristics of the specimen utilized for the treatment design.

Comprehensive genomic profiling (CGP) results		
General Information	Value	
Sex	Male	
Test	Caris MI Profile	
Date	August 2025	
Specimen	Lymph nodes of head, face and neck	
Biomarker	Value	
TMB	11 muts/Mb	
Microsatellite status	MSS	
gLOH	17% (high)	
HRD-GSS (gLOH + LST)	22 (negative)	
Tumor fraction	NA	
Immunohistochemistry	Score	Interpretation
AR	0	Negative
ERBB2 (Her2/Neu)	0	Negative
p16	0	Negative
PD-L1	0%	Negative
Gene	Alteration	VAF (%) or Copies
*CDKN2A*	c.18delG p.S7fs	65
*SMARCA4*	c.1756A>T p.K586*	47
*TP53*	c.743G>T p.R248L	49
*PIK3CA* (VUS)	c.436G>A p.V146I	33
RNA Expression (Selected)	Percentile	Interpretation (≥80 or ≤20)
*AURKA*	89.5	Overexpressed
*CCND1*	1.5	Underexpressed
*CD274* (PD-L1)	79	NA
*CDK4*	96.5	Overexpressed
*CDK6*	13.5	Underexpressed
*CDKN2A*	86.5	Overexpressed
*CTLA4*	85.5	Overexpressed
*EZH2*	93	Overexpressed
*PDCD1LG2*	72	NA
*RB1*	14.5	Underexpressed
*SMARCA2*	6	Underexpressed
*SMARCA4*	3.5	Underexpressed
*SMARCB1*	89	Overexpressed
*TP53*	68	NA
COSMIC Signatures	Percentage	Etiology
SBS4	36	Tobacco/Smoking
SBS3	30	Defective HR DNA repair
SBS5	24	Unknown
SBS30	10	Defective base excision repair

**Figure 3:**
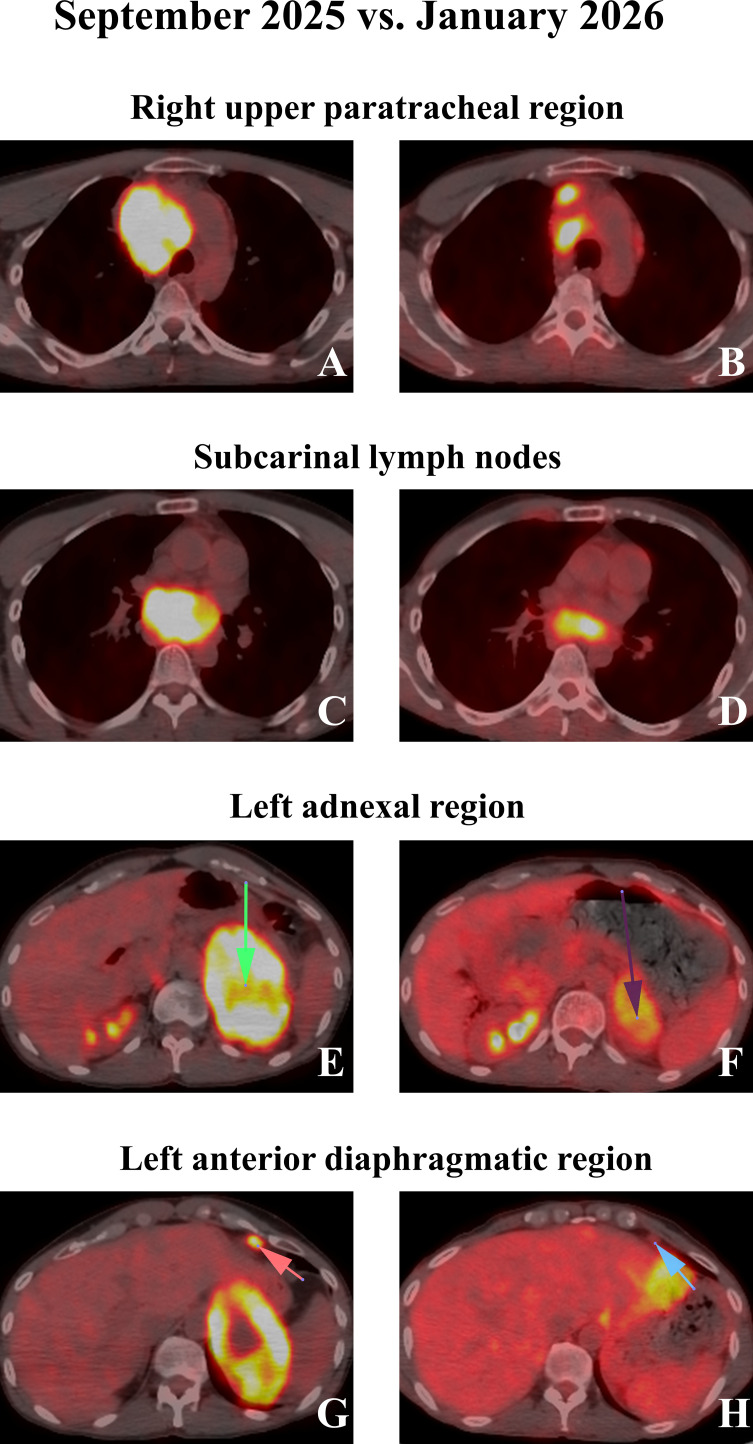
Longitudinal response evaluation. **(A)** Pre-treatment/baseline PET/CT imaging (September 2025) reveals a highly FDG-avid conglomerate lymphadenopathy in the right upper paratracheal region, encasing adjacent vascular structures and causing rightward tracheal deviation. **(B)** Post-treatment scan (January 2026) demonstrates marked regression of the primary mediastinal mass. **(C–H)** Comparative baseline and follow-up imaging highlighting significant anatomic and metabolic regression across multiple metastatic sites, including the subcarinal lymph nodes **(C, D)**, the left adnexal region **(E, F)**, and the left anterior diaphragmatic region **(G, H)**.

Considering the TMB-H status and smoking signature with p16 loss, we initiated immunotherapy plus a CDK4/6 inhibitor combination. We preferred dual immunotherapy over the anti-PD-(L)1 monotherapy due to PD-L1 negativity and high *PDCD1LG2* and *CTLA4* RNA expression (**Figure 2D**, **Table 1**). Concurrent palbociclib was incorporated due to our clinical experience with this specific combination. The dosages were pembrolizumab 100 mg Q4W, ipilimumab 50 mg Q8W, and palbociclib 75 mg/day. The treatment decision was made by a molecular tumor board (MTB), based on the consensus of expert clinicians and molecular biologists. An interim PET/CT scan (January 2026 vs. September 2025, **Figure 3**) demonstrated a significant metabolic regression. During the 14th week, the patient developed grade 4 immune-related hepatitis and grade 2 cardiomyopathy. We discontinued immunotherapy and initiated intravenous methylprednisolone (2 mg/kg). Although liver function tests improved within 48 hours, the patient subsequently experienced ventricular arrhythmias and a reduced ejection fraction, consistent with grade 4 cardiomyopathy. This required management in the intensive care unit (ICU). Due to the steroid-refractory grade 4 cardiomyopathy, abatacept (500 mg IV) was added. The patient responded favorably and was discharged four days later. Following a total of three abatacept doses administered at two-week intervals, the patient’s ejection fraction recovered to 60%. By week 20, the hepatotoxicity and cardiomyopathy had downgraded to grade 2 and grade 1, respectively. Palbociclib therapy is planned to commence once the hepatotoxicity resolves to grade 1.

## Discussion

SMARCA4-UTs constitute a distinct entity of aggressive neoplasms that disproportionately affect middle-aged males. They are identified at advanced stages with frequent metastases to the adrenal glands and lymph nodes, and strongly correlate with a smoking history. Furthermore, SMARCA4-UT demonstrates significant resistance to chemotherapy, with low objective response rates and poor prognosis. Immunotherapy-based regimens have recently yielded favorable outcomes but predictive biomarkers are yet to be explored.

The effect of *SMARCA4* alterations/deficiency toward the immunotherapy efficacy has not been clearly defined as it could be context- and cancer type-dependent. For instance, biallelic *SMARCA4* mutations in a pan-cancer study were associated with resistance to both ICIs and platinum-based regimens in certain tumor types (10). Conversely, monoallelic status in uterine cancer was a positive predictor for ICI efficacy. The monoallelic *SMARCA4*-mutant tumors exhibit a significantly higher TMB compared to the wild-type and biallelic tumors (10.5 vs. 4.4 vs. 6.1 muts/Mb) with divergent mutational landscapes across most cancer types. The VAF (47%) for *SMARCA4* K586* mutation here potentially implies a monoallelic status (**Figure 2B**, **Table 1**), and this is consistent with the findings above. Co-mutations, especially *STK11/KEAP1*, could also exacerbate the immunotherapy outcomes in the *SMARCA4*-mutant tumors such as NSCLC. In the SMARCA4-UT, patients receiving immune-based therapies had longer PFS (8.1 vs. 3.0 months) and OS (14.1 vs. 8.7 months) compared to those treated with non-immunotherapy alternatives (1). We observed a remarkable metabolic response at the first PET/CT evaluation, and it has been ongoing for the last 4 months (**Figure 3**). The Caris CGP reported a tobacco/smoking signature (SBS4 36%, **Table 1**), which was linked to better pathological response to neoadjuvant immunotherapy and superior to PD-L1 expression in lung cancer (21). Expectedly, SMARCA4 deficiency was found to correlate with genomic smoking signature (22). Interestingly, *SMARCA4* mutations, especially the class 1, were more likely to be PD-L1-low or negative but have higher TMB as compared with the WT cases (9). In line, our patient harbored a class I *SMARCA4* mutation leading to protein loss, SBS4 smoking signature (36%) with a heavy smoking history but was negative for PD-L1 (**Figure 2C**, **Table 1**). There are currently no studies comparing the efficacy of single-agent versus dual immunotherapy in the SMARCA4-UT, or in the NSCLC by *SMARCA4* alteration status. However, NSCLC patients with PD-L1-negative tumors have demonstrated a superior long-term survival with dual immunotherapy compared to monotherapy (5-year OS rates: 16.6% vs. 9.3%) (23).

Beyond immunotherapy, our patient’s molecular profile provided a strong rationale for CDK4/6 inhibition. This decision was supported by *CDKN2A* mutation, leading to negative p16 IHC despite high *CDKN2A* mRNA level, and *CCND1* expression (**Figure 2D**, **Table 1**). While we have access to multiple CDK4/6 inhibitors in Türkiye, we prioritized palbociclib over ribociclib and abemaciclib. Palbociclib has a mean plasma elimination half-life of 28.8 hours and is available in multiple doses and formulations (capsule or tablet). It could be utilized in several alternative dosing schedules, such as 1 day on/1 day off or 2 weeks on/2 week off, in addition to the standard 3−weeks−on/1−week−off regimen (24–26). In our clinical practice, we preferentially use a 75−mg dose in 1 day on/1 day off schedule and observe its efficacy in malignancies other than breast cancer (25, 26). Since *CDKN2A* alterations could reduce immunotherapy benefit in multiple cancer types (27, 28), addition of a CDK4/6 inhibitor could potentially improve survival outcomes (29). Moreover, these inhibitors could reverse gene expression changes and/or augment anti-tumor immunity by enhancing T-cell activation in the immunotherapy non-responsive tumor models (30, 31). CDK4/6 inhibitors could induce viral mimicry and cellular senescence in tumor cells that cause senescence-associated secretory phenotype (SASP) and enhance antigen presentation to stimulate cytotoxic T cells, and suppress the proliferation of immunosuppressive Tregs, which could be more dependent on CDK4/6 activity than CD8+ cells (32). Furthermore, we have treated multiple patients with advanced cancers using the exact dual ICI plus palbociclib combination, and we are therefore more familiar with this combination with respect to both efficacy and toxicity management than with immunotherapy regimens incorporating other CDK4/6 inhibitors.

Pembrolizumab is commonly associated with fatigue, diarrhea, anorexia, nausea, musculoskeletal pain, rash, and hypothyroidism. Although mild-moderate aminotransferase elevations may occur in up to 20% of patients (33), a systematic review of pembrolizumab monotherapy clinical trials reported low rates of grade ≥3 AST/ALT elevation (2.26%/1.29%), hepatitis (0.89%), pneumonitis (0.8%), and colitis (0.7%) (34). Furthermore, pembrolizumab is typically administered at 200 mg every 3 weeks or 400 mg every 6 weeks; in our case, 100 mg every 4 weeks was used. Similarly, ipilimumab commonly causes fatigue, diarrhea, dermatologic toxicity, nausea/vomiting, and headache (35), with hepatitis reported in 1-7% during ipilimumab monotherapy while it remarkably increases with combination immunotherapy (13-30%, including 6-19% grade ≥3) (36). Ipilimumab toxicities are dose-dependent (37); therefore, we used 50 mg every 8 weeks, substantially lower than standard weight-based regimens. Cardiotoxicities observed after pembrolizumab or ipilimumab treatment could be severe and potentially fatal even though they were infrequently reported (38). On the other hand, palbociclib predominantly causes reversible and manageable hematologic toxicity, primarily neutropenia (39) and has comparatively favorable hepatotoxicity/cardiotoxicity profiles among the CDK4/6 inhibitors, despite similarly being metabolized in the liver (40–42). In our case, the grade 4 hepatitis and cardiomyopathy were most consistent with immune-mediated toxicity and improved after methylprednisolone (± abatacept). While a contribution from palbociclib cannot be excluded given treatment discontinuation, we anticipate that similar events would be uncommon with dose reduction and/or extended dosing intervals (43, 44). The treating physicians should be more cautious about monitoring and management of the overlapping toxicities, such as pneumonitis/ILD, diarrhea, rash, and fatigue. Even when adverse events are successfully resolved, such as with high-dose corticosteroids in case of immune-related AEs, treatment interruptions or discontinuations during the management period may result in disease progression and, in some cases, death prior to treatment reinitiation. Steroid-related complications, such as infection and hyperglycemia, may deteriorate the patient’s clinical status; therefore, corticosteroids could be administered at the lower doses whenever clinically appropriate (45).

Overall, immunotherapy and targeted agent combinations hold the potential to significantly enhance patient outcomes in the SMARCA4-UT. This is the first instance of a partial response achieved via dual ICB plus palbociclib in a PD-L1-negative, p16-negative, and TMB-high SMARCA4-UT. It underscores the significance of the MTBs in guiding clinical decision-making by integrating diverse data modalities.

## Methods

### Next-generation sequencing

The patient’s mutation and RNA expression data were obtained using the Caris MI Profile^®^ assay. This platform integrates whole-exome sequencing for comprehensive genomic profiling and whole-transcriptome sequencing for RNA profiling. The technical aspects of this proprietary assay have been published by Caris Life Sciences (Irving, TX, USA) (46).

### Immunohistochemistry

Immunohistochemistry for SMARCA4/BRG1 (Abcam, clone ab110641) and pan-Cytokeratin (Biocare, clone AE1/AE3) was performed on 3−µm sections of formalin−fixed, paraffin−embedded (FFPE) tissue using an automated immunostaining platform (Ventana Medical Systems BenchMark XT/ISH staining module). Loss of nuclear SMARCA4/BRG1 expression was assessed, with accompanying lymphocytes serving as an internal positive control. For pan-Cytokeratin, cytoplasmic and membranous staining was considered positive.

## Data Availability

The original contributions presented in the study are included in the article/supplementary material. Further inquiries can be directed to the corresponding authors.
